# SerpinB2 (PAI-2) Modulates Proteostasis via Binding Misfolded Proteins and Promotion of Cytoprotective Inclusion Formation

**DOI:** 10.1371/journal.pone.0130136

**Published:** 2015-06-17

**Authors:** Jodi A. Lee, Justin J. Yerbury, Natalie Farrawell, Robert F. Shearer, Patrick Constantinescu, Danny M. Hatters, Wayne A. Schroder, Andreas Suhrbier, Mark R. Wilson, Darren N. Saunders, Marie Ranson

**Affiliations:** 1 School of Biological Sciences, University of Wollongong, Wollongong, NSW, Australia; 2 Illawarra Health and Medical Research Institute, University of Wollongong, Wollongong, NSW, Australia; 3 Kinghorn Cancer Centre and Cancer Research Program, Garvan Institute of Medical Research, Sydney, NSW, Australia; 4 St Vincent’s Clinical School, Faculty of Medicine, University of New South Wales, Sydney, NSW, Australia; 5 Department of Biochemistry and Molecular Biology and Bio21 Molecular Science and Biotechnology Institute, The University of Melbourne, Melbourne, Vic., Australia; 6 QIMR Berghofer Medical Research Institute, Brisbane, Qld, Australia; UMCG, NETHERLANDS

## Abstract

SerpinB2 (PAI-2), a member of the clade B family of serine protease inhibitors, is one of the most upregulated proteins following cellular stress. Originally described as an inhibitor of urokinase plasminogen activator, its predominant cytoplasmic localisation suggests an intracellular function. SerpinB2 has been reported to display cytoprotective properties in neurons and to interact with intracellular proteins including components of the ubiquitin-proteasome system (UPS). In the current study we explored the potential role of SerpinB2 as a modulator of proteotoxic stress. Initially, we transiently transfected wild-type SerpinB2 and SerpinB2-/- murine embryonic fibroblasts (MEFs) with Huntingtin exon1-polyglutamine (fused C-terminally to mCherry). Inclusion body formation as result of Huntingtin aggregation was evident in the SerpinB2 expressing cells but significantly impaired in the SerpinB2-/- cells, the latter concomitant with loss in cell viability. Importantly, recovery of the wild-type phenotype and cell viability was rescued by retroviral transduction of SerpinB2 expression. SerpinB2 modestly attenuated Huntingtin and amyloid beta fibril formation in vitro and was able to bind preferentially to misfolded proteins. Given the modest chaperone-like activity of SerpinB2 we tested the ability of SerpinB2 to modulate UPS and autophagy activity using a GFP reporter system and autophagy reporter, respectively. Activity of the UPS was reduced and autophagy was dysregulated in SerpinB2-/- compared to wild-type MEFs. Moreover, we observed a non-covalent interaction between ubiquitin and SerpinB2 in cells using GFP-pulldown assays and bimolecular fluorescence complementation. We conclude that SerpinB2 plays an important role in proteostasis as its loss leads to a proteotoxic phenotype associated with an inability to compartmentalize aggregating proteins and a reduced capacity of the UPS.

## Introduction

SerpinB2, also known as plasminogen activator inhibitor type 2 (PAI-2), is expressed by numerous cell types either constitutively (e.g. keratinocytes, peritoneal macrophages, syncytial trophoblasts) or inducibly following inflammation, infection or injury (e.g. monocytes/macrophages, fibroblasts, endothelial cells) [1,2]. Dysregulated SerpinB2 expression and SerpinB2 polymorphisms have been associated with a number of diseases including pre-eclampsia, asthma, periodontitis, lupus, and scleroderma [1,2]. SerpinB2 expression in tumours has also been associated with improved cancer prognosis [3,4]. SerpinB2 is secreted (albeit inefficiently) as a glycosylated protein (~60 kDa) [5] and has classically been thought of as an inhibitor of urokinase plasminogen activator (uPA), and to a lesser extent tissue plasminogen activator (tPA) [6]; the original concept that SerpinB2 is uninvolved in fibrinolysis is not supported by studies in SerpinB2^-/-^ mice [7]. However, SerpinB2 usually exists as a 47 kDa intracellular protein and a number of intracellular binding partners have been reported, including proteasome subunit beta type 1 (PSMβ1, a component of the proteasome), IFN response factor 3, interferon stimulated gene-15 and ZNF198/FGFR1 fusion kinase (reviewed in [1]). These interactions suggest uPA/tPA-independent intracellular role(s) for SerpinB2, although no specific function has yet been determined.

Disruption of normal protein homeostasis (proteostasis), leading to proteotoxic stress, underpins the pathophysiology of inflammation and neuronal dysfunction in acute injury and age-related neurodegeneration [8]. The latter is often associated with protein aggregation as a result of mutations in, for example, amyloid-beta in Alzheimer's disease and huntingtin in Huntington's disease [9]. SerpinB2 is expressed in neurons and microglia and is highly expressed in amyloid-containing microglial conglomerations associated with senile plaques in brain sections from Alzheimer’s disease patients [10]. Moreover, SerpinB2 has been identified as one of the 9 core “Activity-regulated Inhibitor of Death (AID)” genes that mediate neuroprotection by *N*-methyl-D-aspartate (NMDA) receptor-induced calcium signalling [11]. SerpinB2 mRNA levels and expression increased by 180-fold based on microarray data and >1800-fold based on quantitative reverse transcriptase PCR analysis, respectively, within 3 h of injection of the action potential bursting GABA_A_ receptor antagonist bicuculline into the brain [11]. Over-expression of SerpinB2 in the brain was protective against seizure-induced neuronal death (following injection of the glutamate analogue, kainic acid) [11] but the underlying mechanism of protection is not known. SerpinB2 is also one of the most up-regulated proteins in monocyte/macrophages following infection or stimulation with inflammatory mediators, representing up to 0.25% of total intracellular protein under these conditions [12,13]. As other members of the serpin superfamily (e.g. HSP47/SerpinH1) have demonstrated chaperone activity [14], we postulate that intracellular SerpinB2 may function as a stress response protein with cytoprotective activity. In the current study we undertook a series of cellular and biochemical assays to assess this putative function for intracellular SerpinB2.

## Materials and Methods

### Materials

Recombinant human SerpinB2 (47 kDa) was purified from *E*. *coli* (M15) using the pREP4/pQE-9 expression system, as previously described [15]. Bovine serum albumin (BSA), bovine superoxide dismutase (SOD1), creatine phosphokinase (CPK), dithiothreitol (DTT), iodoacetic acid, ovalbumin (SerpinB14), porcine citrate synthase (CS) and Thioflavin-T were all from Sigma-Aldrich. αB-crystallin was a kind gift from Dr Heath Ecroyd (University of Wollongong). Tissue culture supernatant containing antibody against human clusterin from the hybridoma clone "G7" (5–10 ug.mL^-1^) [16] and purified human clusterin from whole blood were prepared as previously described [17]. Amyloid-beta peptide 1–40 and NH_4_OH was from Anaspec. Amyloid-beta 1–42 was from Biopeptide, USA.

### Mouse embryonic fibroblast (MEF) generation

MEFs were isolated from wild-type and SerpinB2^-/-^ mice as previously described [7]. Briefly, the uterine horns containing embryos were dissected from pregnant females that had been euthanised at day 13.5 post coitus by CO_2_ asphyxiation. After removal of each embryo from its amniotic sac, the embryos were homogenised by passing gently through an 18 gauge needle several times. After brief centrifugation of the resulting cell suspension, the cell pellet was resuspended in RPMI-1640 containing 10% fetal calf serum (FSC) and plated out onto 0.1% gelatin (passage 0) and maintained at 37°C with 5% CO_2_. Primary cultures were then continually passaged until spontaneous immortality was obtained. Expression of human SerpinB2 in SerpinB2^-/-^ MEFs was achieved using murine stem cell virus (MSCV)-based pMIG bicistronic viral vector system [18]. This vector contains an internal ribosome entry site (IRES) between the SerpinB2 open reading frame (1.2 kb) and a Green Fluorescent Protein (GFP) reporter, driving expression of both from a single promoter. Viral packaging was performed as described in Brummer *et al* [19]. Briefly, PlatE cells were transfected using Polyfect (Qiagen) and viral supernatants collected after 48 h, then filtered (20μm) and transduction of MEFs performed with polybrene. Transduced cells were selected based on expression of GFP by cell sorting on a FACS Vantage instrument (Becton Dickinson) with the GFP positive population for each transduced cell line ranging from 80–90% of total viable cells. Expression of SerpinB2 was confirmed by RT-PCR and western blot (data not shown). No significant difference in MEF growth characteristics was observed following transduction with either pMIG empty vector or pMIG-SerpinB2 (data not shown).

### Huntingtin exon1 polyglutamine (polyQ) expansion cell model

Huntington's disease is caused by autosomal dominant mutations in the *HTT* gene resulting in expansion of a polyQ sequence near the amino-terminus of the huntingtin (Htt) protein that promotes its aggregation [9]. Wild type alleles of the *HTT* gene encode polyQ sequences that range between 7 and 36 contiguous glutamine residues and mutant Htt is considered to contain >36 and up to 250 contiguous glutamine residues [9]. In the current work we used a well-established Htt aggregation model comprising an exon 1 fragment fused to either 25 polyQ or 46 polyQ sequences [20–22]. While longer expansions are more pathogenic, we have previously demonstrated that aggregation of the 46 polyQ Htt exon 1 fusion protein occurs in an experimentally tractable timeframe in a process that is well characterised [20].

MEFs were seeded into 6-well plates (in triplicate) containing 19 mm coverslips (2x10^5^ cells per well) 24 h before transfection with 200 ng of pcDNA3.1-mCherry (control) or pcDNA3.1-Huntingtin exon1-polyglutamine variants fused C-terminally to mCherry (Htt_ex1_25Q-mCherry or Htt_ex1_46Q-mCherry) expression vectors [21,22], using Lipofectamine2000 (Invitrogen) as per manufacturer’s instructions. At 24, 36 and 48 h post-transfection, cells were harvested by brief trypsinisation, washed in phosphate buffered saline (PBS) and then analysed by flow cytometry (Becton Dickinson). Cells were gated to select those positive for either mCherry alone (marker for Httex1polyQ expression) or for mCherry and GFP (surrogate for SerpinB2 expression in pMIG transduced cells described above) (Figure A in S1 Fig) and viability of these populations then determined by SytoxRed exclusion (Figure B in S1 Fig). Data were normalised against the relevant (mCherry only) control populations to correct for potential bias from differential transfection efficiency.

### Huntingtin *in vitro* aggregation assay

Monomeric Htt_ex1_-46Q-Cerulean-MBP fusion protein was aggregated as previously described [22]. Briefly, Htt_ex1_-46Q-Cerulean-MBP was thawed from frozen stocks, diluted to 9 μM, and rapidly mixed with a 1/20 stock of TEV protease. After 10 min Htt_ex1_-46Q-Cerulean was diluted, with or without SerpinB2, to a final concentration of 2 μM and at a 1:1 final molar ratio of SerpinB2: Htt_ex1_-46Q-Cerulean. Duplicate samples were taken at intervals and the total protein determined by cerulean fluorescence (43,000 M^-1^.cm^-1^ at 434 nm). Samples were centrifuged at 13,000 × g for 30 min and supernatant collected as soluble fraction.

### Huntingtin binding assay

Aggregate ligand blots were performed as previously described [17]. Htt_ex1_-46Q-Cerulean (1 μg) was spotted on nitrocellulose membrane and then blocked with 1% (w/v) heat denatured casein in PBS buffer. SerpinB2 (10 μg/ml solution) or control proteins SerpinB14 (10 μg/ml solution) and clusterin (10 μg/ml solution) were then incubated with separate membranes for 2 h at RT. Bound interactors were detected using antibodies to either SerpinB2 (1:1000; Abcam, UK; ab137588), SerpinB14 (1:30000; Sigma-Aldrich, C6534), or α-clusterin (neat hybridoma culture supernatant) and subsequent HRP conjugated secondary antibodies (1:2000) all diluted in 1% (w/v) heat denatured casein in PBS buffer.

### Denatured protein ligand binding ELISA

Denatured protein binding assays were conducted as previously described [16]. Briefly, CPK or CS in PBS (250 μg.ml^-1^) were incubated overnight in 96-well plates at 4°C (control) or 43°C. BSA (1 mg.ml^-1^) was incubated overnight at 37°C in the absence (control) or presence of 20 mM DTT (250 μg.ml^-1^), followed by incubation with 5 mM iodoacetic acid in PBS at 37°C for 1 h to acetylate free cysteines. After washing to remove unbound proteins and a blocking step, the wells were incubated with either a serial dilution of SerpinB2 (6.25–250 μg.ml^-1^, 1.3 nM–5.3 μM) or a fixed concentration of SerpinB14 (250 μg.ml^-1^, 5.5 μM; negative control) or clusterin (50 μg.ml^-1^, 800 nM; positive control) for 1.5 h at 37°C. Bound interactors were detected using antibodies to either SerpinB2 (1:1000), SerpinB14 (1:30000), or α-clusterin (25 μl.well^-1^ hybridoma culture supernatant) and subsequent HRP conjugated secondary antibodies (1:2000) all diluted in 1% (w/v) heat denatured casein in PBS buffer. Background controls were subtracted from all values.

### Protein aggregation assays

BSA (1 mg/ml) in the absence or presence of a weight to weight ratio of either SerpinB2, αB-crystallin (positive control) or SerpinB14 (negative control) [23] were prepared in filtered PBS containing 20 mM DTT, 0.02% NaN_3_, (pH 7.4) then aliquoted into a 96-well plate (in triplicate) and incubated at 37°C overnight. Turbidity measurements (A_360_) were acquired at 5 min intervals using a FLUOstar Optima plate reader (BMG Labtech, Germany). Background absorbance (PBS containing 20 mM DTT, 0.02% NaN_3_, pH 7.4) was subtracted from all values.

### Amyloid aggregation assays

Amyloid beta_1–40_ and beta_1–42_ peptides (Aβ_1–40_ and Aβ_1–42_, respectively) were prepared as previously described [17,24]. Aβ peptide aggregation was monitored by thioflavin-T fluorescence (25 μM in PBS) in the absence or presence of SerpinB2, αB-crystallin or SOD1 (2.5 μM), as described [24]. Background fluorescence (PBS containing thioflavin-T only) was subtracted from all values.

### Electron microscopy

Aβ_1–42_ peptide (25 μM) was incubated for 12 h at 37°C in the absence or presence of SerpinB2, αB-crystallin or SOD1 (2.5 μM) and samples from each reaction resuspended at 0.1 mg/ml in PBS. An aliquot (2 μl) of each sample was blotted onto formvar resin (with carbon coating) nickel mesh grids, incubated for 5 min at RT, washed three times with filtered dH_2_O and negatively stained with 1% uranyl acetate. Specimens were visualised using a JSM7500FA cold Field Emission Gun Scanning Electron Microscope (JEOL) with an acceleration voltage of 20.0 kV, working distance of 8 mm (spot size setting of 8), and transmission electron detector mounted beneath the specimen platform (AIIM Microscopy Facility, University of Wollongong). Secondary electron images were taken with a semi in-lens detector at a working distance of 8.0 mm.

### GFPu Ubiquitin-Proteasome System (UPS) reporter assay

To quantify UPS activity we used the fluorescent UPS reporter GFPu, which relies on a 16 amino acid degron (CL1) fused to the carboxyl terminus of GFP [25,26]. Upon proteasome inhibition the GFP molecule accumulates in the cell, providing a readout on relative UPS activity. GFPu was transfected into MEFs (in triplicate) as above and the level of GFP fluorescence per cell determined using flow cytometry (Becton Dickinson) 48 h after transfection.

### Autophagy reporter assay

To quantify autophagy dysfunction we used the Lyso-ID autophagy reporter (Enzo Life Sciences) as has been used previously [27]. Lyso-ID, an acidic organelle-selective dye, allows the quantification of accumulation of acidic compartments such as those in the autophagy pathway. MEFs were seeded into a 96-well plate 24 h before treatment with an autophagy inhibitor 3-methyladenine (3-MA, 0–5 mM; Adipogen) or an autophagy inducer, verapamil (0–100 μM; Enzo Life Sciences). MEFs were incubated with 3-MA and verapamil overnight (~ 18 h) before quantification using Lyso-ID kit (ENZO). Briefly, cells were washed and incubated with the Lyso-ID red dye for 30 min at RT before measuring the level of red fluorescence per cell on the IncucyteZoom automated imaging system (ESSEN Bioscience). Data shown is corrected for background fluorescence.

### Bimolecular fluorescence complementation (BiFC)

BiFC enables direct visualisation in live cells of protein interactions via the use of two complementary fragments (V1 or V2) of the Venus yellow fluorescent protein—when brought into close proximity by an interaction between proteins fused to these fragments, they refold to form a fluorescent protein [28]. Human SerpinB2 cDNA was cloned into either pcDNA6.2⁄N-EmGFP-DEST (V356-20, Invitrogen) or BiFC vectors (Saunders *et al*, manuscript in preparation), driving expression of GFP-SerpinB2-V5, or V1/V2 fusion proteins, respectively, via recombination cloning. BiFC vectors were also generated containing Ubiquitin (Ub) cDNA as previously described [29]. HEK293T or HeLa cells were seeded onto glass coverslips in 6-well plates at 75,000 cells/well, in DMEM supplemented with 10% FCS 24 h before transfection with 1 μg of plasmid DNA (0.5 μg of each plasmid in the case for BiFC) using X-tremeGENE (Roche), as per manufacturer’s instructions. Coverslips were fixed in 4% (w/v) paraformaldehyde, counterstained with 6-diamidino-2-phenylindole (DAPI) nuclear counter stain (Vector Laboratories, Australia) and analysed using epifluorescence microscopy 24 h post-transfection. Images were captured using a Zeiss Axiocam MRm digital camera Axiovision software V4.8.1.0 (Zeiss). No fluorescence was observed in negative controls including cells transfected with single BiFC expression vectors (data not shown).

In separate experiments, transfected cells were lysed and the identity of specific proteins analysed by immunoblot with antibodies against either SerpinB2 (1:200; #375G, polyclonal goat anti-human PAI-2, American diagnostic Inc, USA), Ub (1:5,000; #KT-76, mouse anti-human Ub (Santa Cruz, USA), GFP (1:5,000; # MMS-118P mouse anti-GFP, Covance, USA; note: specifically recognizes V1 fragment), GFP (1:5,000; # 11814460001 mouse anti-GFP, Roche, USA; note: specifically recognises V2 fragment), and α-tubulin (1:5000; #TU-01, mouse anti-human α-tubulin, Pierce, USA). Ub, GFP and α-Tubulin were detected with rabbit anti-mouse Ig HRP conjugate (1:10000; Sigma-Aldrich, Australia). SerpinB2 was detected with donkey anti-goat Ig HRP conjugate (1:5000; Sigma-Aldrich, Australia).

### GFP-trap assay

HEK293T cells were transfected with GFP or GFP-SerpinB2 expression vectors described above and lysed 24 h later. Soluble protein was collected (lysate) and 40 μg of total protein subjected to affinity purification using GFP affinity resin (GFP-Trap, Chromatek, USA) as per the manufacturer’s instructions.

### Statistical Analyses

Unless stated otherwise, data and statistical analyses were performed using the multiple t test analysis function on GraphPad Prism (version 6.00 for Windows, GraphPad Software, La Jolla California USA, ww.graphpad.com). Statistical significance was determined using the Holm-Sidak method for pairwise comparisons. *P* values < 0.05 were considered statistically significant.

## Results

### SerpinB2 protects cells from Httex1-polyQ expansion-induced toxicity

**Fig 1:**
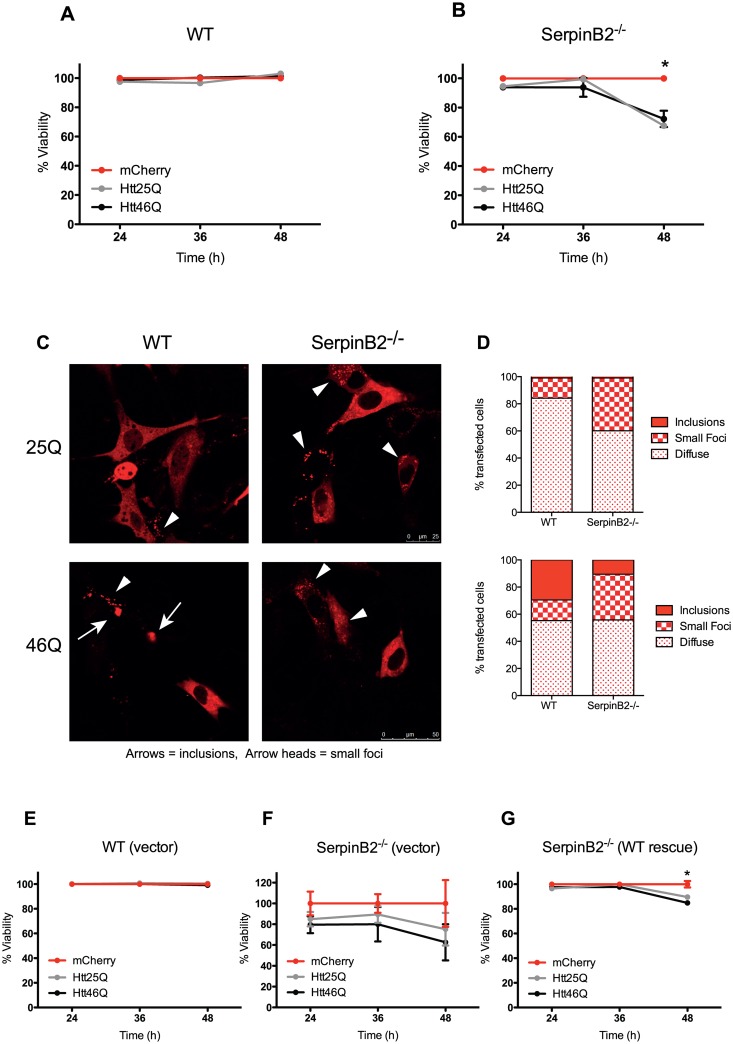
SerpinB2 protects cells from Htt Exon1 polyQ expansion-induced toxicity. **A, B.** Viability of wild-type (A) or SerpinB2^-/-^ (B) MEFs following transient transfection with Htt_ex1_25Q-mCherry, Htt_ex1_46Q-mCherry, or mCherry expression alone (control) vectors. Data represent mean percentage of viable cells (as measured by SytoxRed exclusion and flow cytometry) normalised to mCherry only controls (n = 3 ± SEM). * Htt25q and Htt46q values significantly different from mCherry at 48 h post transfection, *P* < 0.01; **C.** Inclusion formation in wild-type versus SerpinB2^-/-^ MEFs 48 h post transfection with either Htt_ex1_25Q-mCherry or Htt_ex1_46Q-mCherry. Two types of Htt foci were observed, small (< 2um; white arrow heads) and large (> 2 um; white arrows); **D.** Distribution of Htt in MEFs was quantified as diffuse, small foci, or inclusion in each cell; **E, F, G.** Viability of wild-type (WT) (E) or SerpinB2^-/-^ (F) MEFS transduced with pMIG control empty vector (vector), or SerpinB2^-/-^ MEFS transduced with pMIG-SerpinB2 vector (WT rescue) (G) following transfection with Htt_ex1_25Q-mCherry, Htt_ex1_46Q-mCherry or mCherry expression vectors. Data represent mean percentage of viable cells normalised to mCherry only controls (n = 3 ± SEM). * Htt46Q value significantly different from mCherry at 48 h post transfection, *P* = 0.011.

Htt polyQ expansion is characterized by the aggregation of Htt and decreased cell survival in a polyQ-expansion dependent manner in neuronal models [30]. Expression of either wild-type Htt_ex1_25Q-mCherry or pathogenic Htt_ex1_46Q-mCherry induced a similar and significant loss (approximately 25%) in viability of SerpinB2^-/-^ MEFs 48 h after transfection, compared to the mCherry alone vector, with little effect on wild-type MEFs observed (Fig 1A and 1B). This is consistent with previous work using wild-type MEFs that showed no difference in toxicity responses between cells expressing Htt_ex1_25Q and Htt_ex1_97Q [31]. The sensitization of cells to overexpression of both wild-type and pathogenic Htt_ex1_polyQ variants in the absence of SerpinB2 suggests a cytoprotective role for SerpinB2.

We next compared the partitioning of Htt in wild-type and SerpinB2^-/-^ MEFs. Previous work has established that overexpression of Htt_ex1_ containing expanded polyQ repeats (including 46Q used in this study) produces a dense round inclusion in the cytoplasm or nucleus [21,32], while wild-type or 25Q expansion does not. Following expression of Htt_ex1_25Q-mCherry in wild-type MEFs we observed small, Htt-positive foci in ~15% of cells (Fig 1C and 1D). These foci were < 2 μm in diameter and were counted separately to inclusions (Fig 1C). The foci were not stress granules as they were not TIA-1 positive (S2 Fig). Co-transfection studies reveal that at least a fraction of these foci co-localized with LC3-GFP suggesting that the foci were autophagosomes (S3 Fig). There was an increase in the proportion of cells containing these small foci (from ~ 15% to ~ 40%) in the absence of SerpinB2 (Fig 1D), indicating a significant shift in localisation of Htt_ex1_25Q in SerpinB2^-/-^ cells. Following expression of Htt_ex1_46Q in wild-type MEFs we observed a large proportion of cells containing inclusions (~ 30%) that were not present in 25Q-expressing cells (Fig 1C and 1D). However, ~ 15% of cells still contained small Htt foci in 46Q-expressing wild-type cells. In contrast, the proportion of cells containing inclusions was reduced and the number of cells containing smaller fluorescent foci increased following expression of Htt_ex1_46Q-mCherry in SerpinB2^-/-^ MEFs (Fig 1C and 1D). Immunostaining for SerpinB2 in wild-type cells expressing Htt_ex1_46Q-mCherry failed to detect SerpinB2 in inclusions (S4 Fig), indicating that SerpinB2 does not become sequestered by Htt inclusions.

Taken together, the results above suggest that SerpinB2 plays some role in modulating Htt aggregation and provides some protection from protein overexpression or proteotoxic stress. To further address the specificity of this effect in SerpinB2^-/-^ MEFs we performed rescue experiments using retroviral transduction to introduce SerpinB2 in the Htt_ex1_-polyQ expansion- model. Empty vector (i.e. pMIG-GFP) viral transduction of wild-type MEFs [WT (vector)] had no effect on cell viability following expression of Htt_ex1_25Q-mCherry or Htt_ex1_46Q-mCherry (Fig 1E). However, expression of Htt_ex1_25Q-mCherry or Htt_ex1_46Q-mCherry in SerpinB2^-/-^ MEFs previously transduced with empty pMIG-GFP vector [SerpinB2^-/-^ (vector)] induced decreases in cell viability over time with the 46Q variant tending to cause greater losses in cell survival compared to 25Q variant, though this effect did not reach significance (Fig 1F). Re-expression of SerpinB2 in SerpinB2^-/-^ MEFS [SerpinB2^-/-^ (WT rescue)] rescued the deleterious effect of transient Htt_ex1_25Q-mCherry or Htt_ex1_46Q-mCherry expression up to 36 h post-transfection (Fig 1G; no significant differences observed between Htt variants). By 48 h post-transfection there was a small decrease in cell viability caused by expression of both Htt variants compared to the mCherry vector, though this reached significance only for the Htt46Q variant (Fig 1G).

We also noted that when normalized to a transfection reagent only control that the transduced SerpinB2^-/-^ MEFs [SerpinB2^-/-^ (vector)] were also highly susceptible to the deleterious effect of transient mCherry expression (regardless of Htt variant expression) (S5 Fig). This was in stark contrast to both the non-transduced SerpinB2^-/-^ MEFs or the transduced SerpinB2^-/-^ MEFS with WT rescue in which mCherry alone had no deleterious effect on cell viability compared to the transfection reagent only control (S5 Fig). As expected, WT MEFS were completely unaffected by transient transfection with mCherry alone (data not shown). This clearly illustrates the sensitivity of SerpinB2^-/-^ cells to any kind of protein overexpression stress further supporting a role for SerpinB2 in cellular protection against proteotoxicity.

### SerpinB2 attenuates Htt46Q fibril formation *in vitro*.

**Fig 2:**
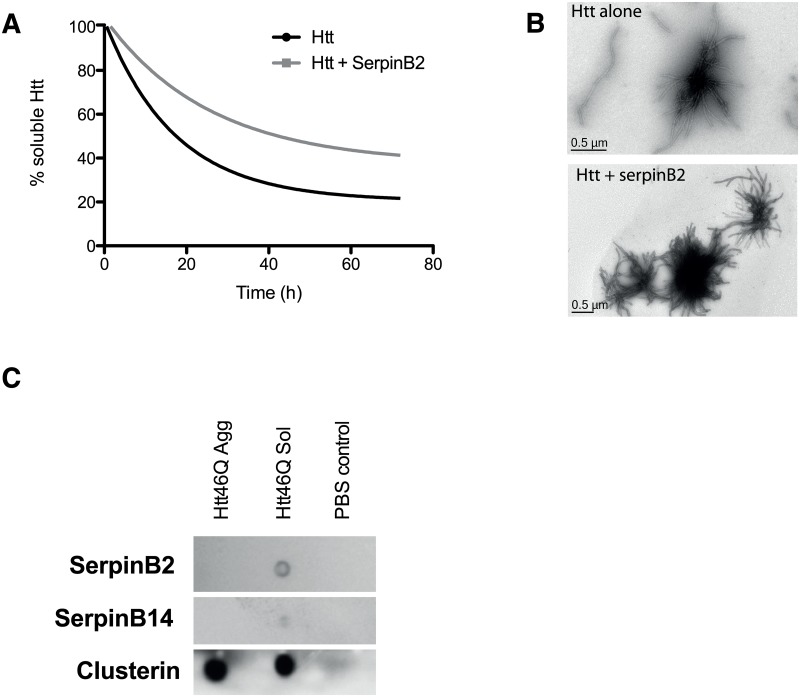
SerpinB2 attenuates Htt42q fibril formation *in vitro*. **A.** Soluble Htt remaining in Htt_ex1_46Q-Cerulean aggregation reactions in the absence or presence of SerpinB2 (equimolar ratio); **B.** TEM images of fibril formation at 72 h; **C.** Representative ligand blot (n = 3) showing differences in binding of SerpinB2 to soluble and aggregated Htt_ex1_46Q. Binding was determined by immunoassay. SerpinB14 or clusterin binding to soluble and aggregated Htt_ex1_46Q are also shown.

Given that SerpinB2 expression can drive changes in protein inclusion formation and cell viability, we tested the ability of recombinant SerpinB2 to suppress *in vitro* fibril formation by Htt_ex1_46Q fused to the monomeric cyan fluorescent protein Cerulean. To prevent aggregation during recombinant protein production, Htt_ex1_46Q-Cerulean was expressed as a fusion protein with a cleavable MBP tag. As previously reported [20], the Htt_ex1_46Q-Cerulean formed insoluble, fibrillar aggregates over 72 h following cleavage with TEV protease to remove the MBP tag (Fig 2A). The presence of recombinant SerpinB2 modestly attenuated the amount of insoluble Htt_ex1_46Q-Cerulean but the morphology of Htt_ex1_46Q-Cerulean insoluble fibrils was indistinguishable in the presence or absence of SerpinB2 (Fig 2B). To examine if the modest attenuation of fibril formation was due to interaction of SerpinB2 with Htt_ex1_46Q-Cerulean, a solid phase ligand-blotting assay was used [17]. SerpinB2 bound to non-aggregated—but not aggregated—Htt_ex1_46Q-Cerulean (Fig 2C). Furthermore, the closely-related SerpinB14 displayed only limited or no binding to non-aggregated Htt_ex1_46Q-Cerulean, or aggregated Htt_ex1_46Q-Cerulean, respectively (Fig 2C). The established extracellular chaperone clusterin [17] bound to both insoluble and soluble Htt_ex1_46Q-Cerulean (Fig 2C).

### SerpinB2 prevents beta-amyloid fibril formation *in vitro*

**Fig 3:**
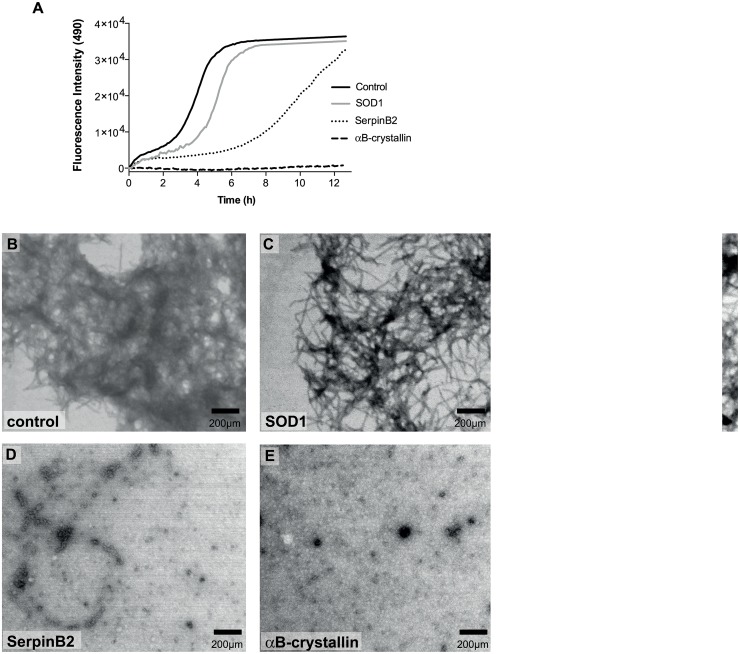
SerpinB2 prevents beta-amyloid fibril formation *in vitro*. **A.** Aβ_1–42_ aggregation was followed by changes in thioflavin-T fluorescence (490 nm) over time in the absence (control) or presence of SerpinB2, SOD1 (negative control) or αB-crystallin (positive control) at a 1:10 final molar ratio of proteins:Aβ_1–42_. Data represent mean fluorescence intensity with background controls subtracted (n = 2; representative experiment shown); **B–E**. Electron microscopy images of end point aggregates from Aβ_1–42_ incubated alone (B), with SOD1 (C), SerpinB2 (D), or αB-crystallin (E). (scale bar = 200μm).

Given the observation of SerpinB2 in senile plaques of Alzheimer’s patients, and other published works showing interactions between amyloid-β and various serpins (including SerpinB2) [33,34], we next investigated the ability of SerpinB2 to inhibit fibril formation by the amyloid-β peptide (Aβ). SerpinB2 was able to completely inhibit aggregation of Aβ_1–40_ peptide at a 10:1 molar ratio of Aβ_1–40_:SerpinB2 (S6 Fig). However, SerpinB2 was not as efficient as αB-crystallin, a well-characterised chaperone [35], at inhibiting the faster aggregating Aβ_1–42_ peptide (Fig 3A). SerpinB2 was able to inhibit Aβ_1–42_ aggregation during the first 5 h of incubation (as seen by the extended lag phase), but this effect was not sustained, as a substantial increase in thioflavin-T fluorescence was observed in the latter part of the assay (Fig 3A). In comparison, αB-crystallin completely inhibited Aβ_1–42_ aggregation for the duration of the assay (Fig 3A). SOD, used as a standard negative control in these assays, had minimal effect on either Aβ_1–40_ (S6 Fig) or Aβ_1–42_ peptide aggregation (Fig 3).

As a complementary approach to analyse the ability of SerpinB2 to inhibit Aβ peptide fibril formation, we performed electron microscopy analysis of samples taken from Aβ_1–42_ solutions in the presence or absence of 10-fold molar excesses of SerpinB2, aβ-crystallin or SOD, following incubation for 12 h at 37°C. Aβ_1–42_ peptides formed fibrils (Fig 3B) under these conditions. SOD was unable to prevent fibril formation (Fig 3C). In contrast, both SerpinB2 (Fig 3D) and αB-crystallin (Fig 3E) completely inhibited fibril formation.

### SerpinB2 binds to stress induced misfolded proteins and attenuates aggregation *in vitro*

**Fig 4:**
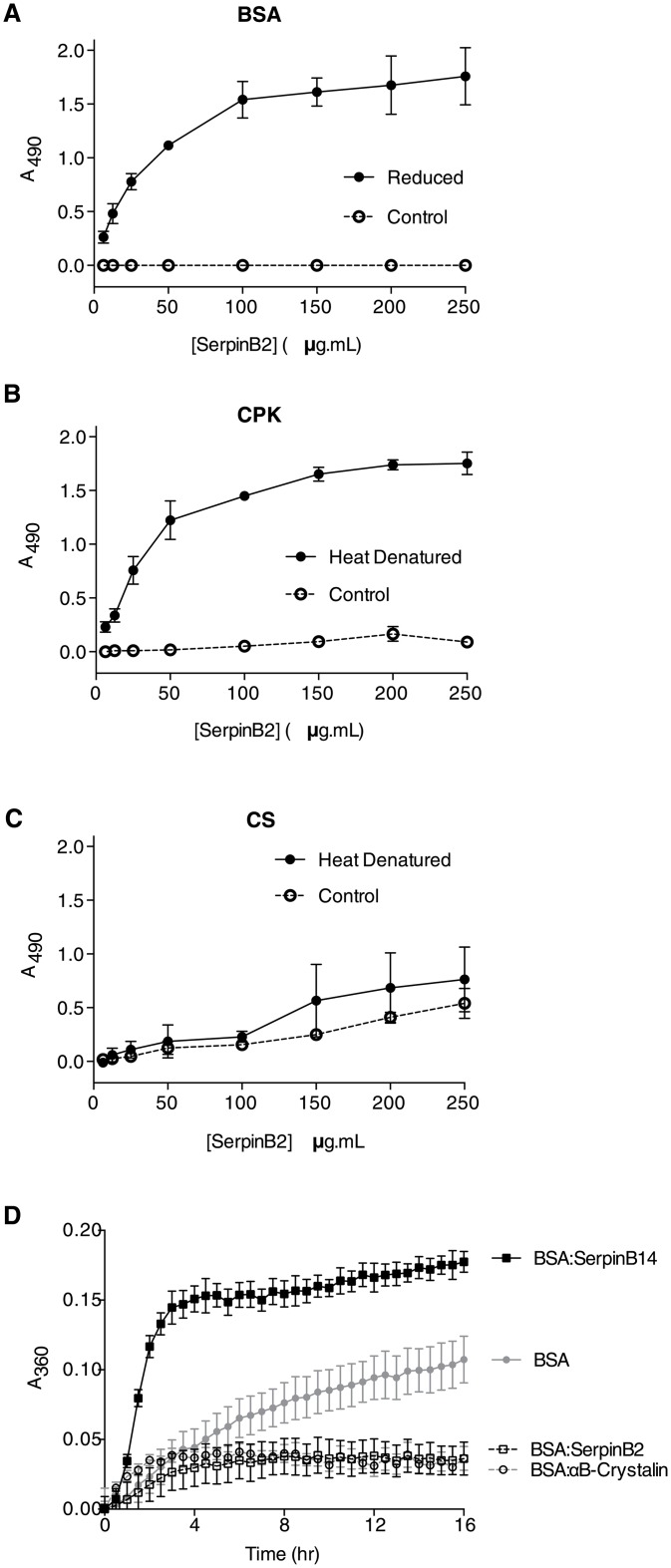
SerpinB2 binds misfolded proteins and suppresses BSA aggregation *in vitro*. **A–C.** Dose dependent binding of SerpinB2 to native and misfolded proteins was determined using ELISA. Data represent mean absorbance (A_490_) ± SEM (n = 3); **D.** Real-time turbidity assay of DTT-induced BSA aggregation in the absence or presence of SerpinB2, αB-crystallin or SerpinB14 (w/w ratio). Data represent mean absorbance (A_490_) ± SEM (n = 3).

A defining characteristic of chaperones is their ability to bind to misfolded or unfolded proteins, usually via hydrophobic interactions [36–39]. We first investigated the ability of SerpinB2 to bind misfolded proteins using ELISA. SerpinB2 showed saturable and specific binding to reduction (DTT)-stressed BSA (Fig 4A) and heat-stressed CPK (Fig 4B) but negligible binding to native proteins was detected. As expected, the chaperone clusterin also bound to misfolded BSA (data not shown). SerpinB2 bound at low levels to both native and heat-stressed CS (Fig 4C). The related (non-inhibitory) clade B serpin, SerpinB14, did not appreciably bind native or misfolded forms of BSA, CPK or CS (data not shown).

Given that SerpinB2 binds specifically to misfolded BSA we next investigated the ability of SerpinB2 to inhibit the stress-induced aggregation of this client protein. SerpinB2 inhibited the DTT-induced aggregation of BSA to the same extent as αB-crystallin (Fig 4D). In contrast, SerpinB14 enhanced DTT-induced BSA aggregation (Fig 4D). Neither αB-crystallin nor serpins alone aggregated under the reducing conditions of this assay (data not shown).

### SerpinB2 regulates cellular proteostasis and binds to ubiquitin non-covalently

**Fig 5:**
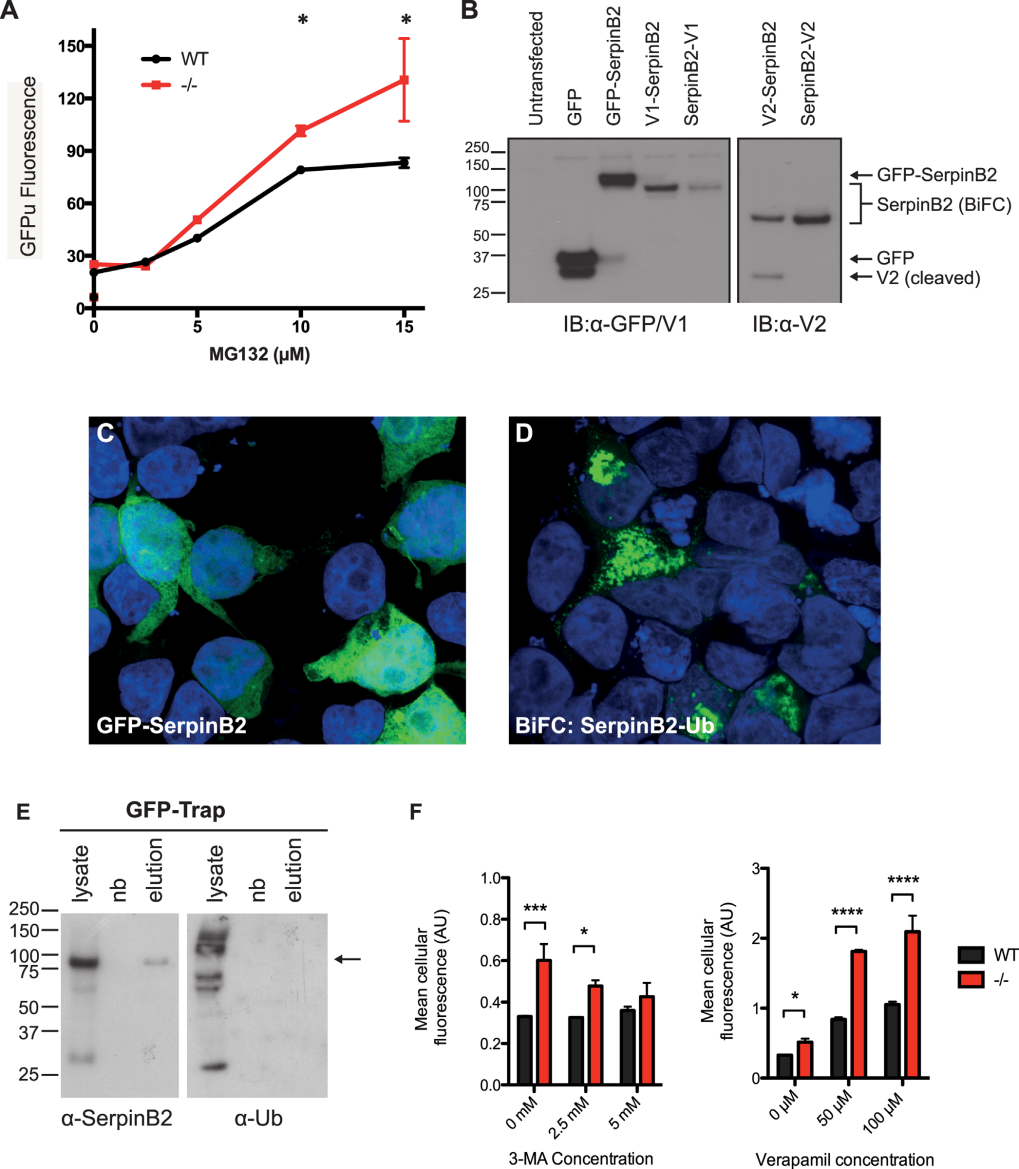
SerpinB2 modulates UPS activity and binds Ubiquitin. **A.** Dose response of UPS activity (GFPu fluorescence) in wild-type (WT) and SerpinB2^-/-^ MEFs after 24 h treatment with proteasome inhibitor MG132. Note that fluorescence levels prior to addition of MG132 were similar in both cell types indicating similar degree of transfection with GFPu plasmid. Data represent mean fluorescence (_515_20 blue_) ± SEM (n = 3). * Values for SerpinB2^-/-^ MEFS significantly different from wild-type cells at the corresponding MG132 concentration, *P* < 0.01; **B.** Immunoblot analysis validating correct expression of GFP and BiFC fusion proteins. GFP-SerpinB2, V1-SerpinB2 and SerpinB2-V1, and V2-SerpinB2 and SerpinB2-V2 were detected at the expected combined molecular weights of ~ 75 kDa, ~ 60 kDa (V1 is ~ 13 kDa), and ~ 55 kDa, (V2 is ~ 8 kDa), respectively; **C.** Epifluorescence microscopy showing GFP-SerpinB2 expression (green) in HEK293T cells, cell nuclei stained with DAPI (blue); **D.** Confocal microscopy showing BiFC signal (green) indicating SerpinB2 interaction with Ubiquitin (Ub) in HEK293T cells, cell nuclei stained with DAPI (blue). Scale bars = 20μm; **E.** GFP-SerpinB2 pull down and immunoblot analysis. GFP-SerpinB2 expressing HEK 293T cells were lysed (lysate) and samples applied to GFP affinity resin. Aliquots from the lysate and non-bound (nb) and eluted (elution) fractions (~ 40 μg) were analysed by immunoblot using antibodies against either SerpinB2 (α-SerpinB2) or Ub (α-Ub) as shown. Arrow indicates position of GFP-SerpinB2 in the SerpinB2 blot (left panel). A band of similar size is not present in the Ub blot (right panel). No proteins were detected in the elution fractions derived from untransfected or GFP only expressing cell lysates (data not shown); **F.** Dose response of autophagy activity (cellular associated Lyso-ID fluorescence) in WT and SerpinB2^-/-^ MEFs after 18 h treatment with autophagy inhibitor 3-MA or autophagy inducer verapamil. Data represent mean cellular fluorescence ± SEM (n = 3). **** indicates *P* < 0.0001; *** indicates *P* < 0.001, * indicates *P* < 0.05.

SerpinB2 has previously been found in association with the proteasome subunit PSMβ1 [40,41] and in screening assays for novel Ub binding proteins (Saunders *et al*., unpublished) suggesting that SerpinB2 might bind Ub. Given this information, the chaperone-like activity of SerpinB2 and its apparent ability to modify the partitioning of Htt, and the known interactions between chaperones and the UPS we investigated a potential role for endogenous SerpinB2 in modulating protein degradation pathways, using the GFPu reporter of cellular UPS activity after incubation with the proteasome inhibitor MG132. As expected, we observed a dose-dependent increase in GFPu signal in response to proteasome inhibition by MG132 in both (Fig 5A). This signal was significantly increased in SerpinB2^-/-^ MEFs at MG132 concentrations > 5 μM compared to the wild-type cells (Fig 5A). A dose response analysis of cell viability after 48 h treatment with MG132 revealed half maximal inhibitory concentration values of ~10 μM for both cell lines (data not shown), indicating that toxicity issues were not responsible for this difference. These data suggest that the absence of SerpinB2 in cells places stress on the UPS.

We undertook two separate assays (BiFC and affinity purification) to further investigate the role of SerpinB2 in modulating proteostasis via a potential interaction with Ub. Expression of fusion proteins of expected size (V1-SerpinB2, SerpinB2-V1, V2-SerpinB2, SerpinB2-V2, GFP-SerpinB2 or GFP) following transfection was confirmed by western blotting (Fig 5B). GFP-SerpinB2 was strongly expressed and evenly localised throughout the cytosol and nucleus of transfected HEK293T cells (Fig 5C). Strong BiFC signal was observed in HEK293T cells following co-transfection with V2-SerpinB2 and V1-Ub expression vectors, suggesting an interaction between SerpinB2 and Ub proteins in discrete cellular locations (Fig 5D). Similar BiFC signal levels and distribution patterns were obtained in both HEK293T and HeLa cells following co-transfection with V1- or V2-SerpinB2 and V1-Ub (data not shown).

After BiFC confirmation of a cellular interaction between SerpinB2 and Ub, the nature of the interaction was further investigated using GFP-trap affinity purification from GFP-SerpinB2 expressing cells. SerpinB2-GFP (~ 75 kDa) was detected in whole cell lysates and GFP-trap elution but not in the nonbound fraction (Fig 5E, left panel), indicating efficient capture of GFP-SerpinB2. Immunoblotting for Ub showed the presence of high molecular weight “smears”, representing a range of cytosolic ubiquitinated proteins in whole cell lysate. However, no Ub was detected in the elution fractions (Fig 5E, right panel). Hence, while BiFC data indicate an intracellular interaction between SerpinB2 and Ub, biochemical analysis suggests that this association is non-covalent. That is, SerpinB2 associates with Ub in cells but is not itself ubiquitinated.

The interaction of SerpinB2 with Ub and modulation of Htt_ex1_46Q-mCherry distribution in MEFs by SerpinB2—with an increase in autophagosome structures—prompted us to examine the potential role of SerpinB2 in autophagy. Previous work has suggested a role for SerpinB2 in Beclin-1 autophagy pathways [42]. Wild-type and SerpinB2^-/-^ MEFs were incubated in the absence or presence of the autophagy inhibitor 3-MA or promoter verapamil and the number of autophagosomes quantified using the Lyso-ID kit as previously described [27]. Even in the absence of the autophagy modulators the amount of autophagosome signal (indicated by cellular fluorescence) was significantly increased in SerpinB2^-/-^ MEFs compared to WT cells (Fig 5F), indicating a dysregulation of autophagy pathways. In the presence of 3-MA, a small increase in fluorescence was observed in WT cells at the highest concentration (5 mM) (Fig 5F, left panel). In contrast, while there was a dose-dependent decrease in fluorescence in SerpinB2^-/-^ MEFS with increasing 3-MA concentration (likely due to the stress placed on the cells under these inhibitory conditions), compared to WT cells the level of fluorescence was significantly higher in the presence of 2.5 mM 3-MA. The increase in fluorescence indicates an accumulation of autophagosomes as a result of inhibition of autophagy. Upon verapamil treatment, which induces the rate of autophagosome formation [27], an increase in fluorescence with increasing verapamil concentrations was observed in both WT and SerpinB2^-/-^ MEFS, however fluorescence associated with the latter was significantly increased compared to WT MEFs (Fig 5F, right panel), which is also consistent with dysregulation of autophagy pathways.

## Discussion

Disruption of proteostasis is a key driver in the pathophysiology of inflammation and neuronal dysfunction [8]. In these contexts, significant increases in SerpinB2 expression have been reported. For example, SerpinB2 expression increases rapidly and significantly in acute brain injury models [11] and this overexpression mediates neuroprotection via undefined mechanisms. Further, SerpinB2 is highly expressed in amyloid-containing microglial conglomerations associated with Alzheimer’s senile plaques [10,11]. These observations, and the role of other serpin molecules in modulating proteostasis through chaperone-like activities [14], led us to investigate the potential cytoprotective mechanisms of SerpinB2.

One mechanism by which cells under proteotoxic stress can protect themselves is through the production of large protein inclusions, presumably to sequester toxic smaller oligomeric aggregates that are not visible using conventional confocal microscopy [43]. Previous work has shown that oligomers formed on the amyloid forming pathway are more toxic than the mature aggregates and that this correlates to the exposed hydrophobicity of the aggregates [24]. Cells have evolved specific mechanisms to reduce the exposure of soluble toxic oligomeric species by sequestering misfolded proteins to specialized sites in the cell. For example, proteins containing polyQ expansions (e.g. Htt) have been shown to reliably coalesce into **i**nsoluble **p**rotein **d**eposit (IPOD) inclusions in mammalian cells [21,32,44]. IPOD inclusions are characterized by small dense cytoplasmic inclusions of aggregated protein that accumulate at a distance from the nucleus [21,32,44]. Depending on the cell type Htt IPOD inclusion formation is a predictor of improved survival in time-resolved single cell experiments, decreasing accessibility of mutant Htt elsewhere in the cell [30]. Our observations of decreased numbers of large IPOD inclusions and increased cell death in SerpinB2-null cells (Fig 1) are consistent with a role for SerpinB2 in mediating the formation of cellular inclusions. SerpinB2 does not co-localize with Htt IPOD structures, but this does not preclude an indirect role in inclusion formation. An RNAi genome wide screen in in *C*. *elegans* identified RNA synthesis, protein synthesis, protein folding, protein transport and protein degradation as vital classes of genes that control polyQ protein aggregation [45]. This elegant study implicated all aspects of the proteostasis network as modulators of protein aggregation *in vivo*. Hence, our data showing reduced numbers of large Htt46Q inclusions in the absence of SerpinB2 suggests SerpinB2 is part of the cellular proteostasis regulation network. SerpinB2 does not appear to exert its protective effects by interacting directly with aggregated Htt. While the precise role of SerpinB2 in Htt inclusion formation is still somewhat unclear, a reduction in the capacity of the proteostasis machinery, such as protein degradation, in the cell may shift cellular resources allowing alternate distribution of Htt and result in a vulnerability to protein overexpression related toxicity (regardless of polyQ length) consistent with our observations in this study. Indeed aggregation of Htt has been shown to inhibit the proteasome indirectly by sequestering the chaperone Sis1p and suppressing nuclear degradation in yeast-based studies [46].

Although SerpinB2 had minimal effect on Htt46Q fibril formation in biochemical assays using purified proteins (Fig 2), SerpinB2 significantly inhibited formation of amyloid-β fibrils, similar to the effects of the established chaperone αB-crystallin. Moreover, SerpinB2 bound specifically to misfolded (but not native) proteins and inhibited the reduction-induced amorphous aggregation of BSA *in vitro* (Fig 3). We did not observe these effects using the closely related molecule SerpinB14. These results suggest a chaperone-like function for SerpinB2, which is perhaps not surprising as it is known that other serpins interact with Aβ peptides. Serpins A1 (α1-antitrypsin) [47], A3 (α1-antichymotrypsin) [48,49], and I1 (neuroserpin) [34] have all been identified within senile plaques associated with Alzheimer’s Disease (AD) or in surrounding tissue/supporting stroma. Further, SerpinA3 has been reported to inhibit Aβ_1–40_ fibril formation at a Aβ:serpin ratio of 10:1 [50] and 15:1 [51], though toxicity of Aβ_1–40_ peptide was unaffected [51]. In similar experiments, Aksenov et al. [52] found that SerpinA3 did not inhibit fibril formation using Aβ_1–42_ but did reduce the toxicity of the peptide. These data support the model that prefibrillar oligomers, rather than fibrils, are the primary toxic species [24]. Whether the chaperone-like capacity of SerpinB2 contributes to its cytoprotective functions *in vivo* remains to be determined.

Protein degradation via the UPS and autophagy is a key component of the proteostasis network [53]. Intriguingly, a common SerpinB2 polymorphism is associated with susceptibility to dose limiting peripheral neuropathy in myeloma patients undergoing therapy with the proteasome inhibitor Bortezomib [54]. We showed that loss of SerpinB2 attenuates cellular capacity to degrade the GFPu reporter of UPS activity when the proteasome is partially blocked by MG132. Expression of misfolded proteins (such as Huntingtin) also increases the signal from UPS reporters (implying inhibition of degradation) by sequestering a HSP40 chaperone, which is vital for degradation of cytosolic proteins [46]. In addition, we found that the absence of SerpinB2 modulated the accumulation of autophagosomes in MEFs. Consistent with our observations, previous work has also suggested a role for SerpinB2 in autophagy pathways [42], and Htt aggregation can be manipulated by perturbing autophagy [55].

BiFC and biochemical data suggest that the observed interaction between SerpinB2 and Ub is non-covalent and therefore does not represent classical ubiquitylation of SerpinB2. Many non-covalent interactions with Ub have been reported [56]. In general, proteins which interact non-covalently with Ub contain small regions known as Ub binding domains (UBDs) [57]. UBDs are structurally diverse and can recognise and bind to different length Ub chains attached to proteins [57]. The role of UBDs in mediating biological processes is still poorly understood. However, it is known that UBDs can act as mediators in assisting or repressing proteasome-mediated degradation of ubiquitinated proteins [58]. These proteins can act to regulate degradation of proteasome targeted proteins by transiently binding to the poly-Ub chain, or blocking it’s recognition by the 19S regulatory particle on the proteasome, thereby preventing substrate degradation. Furthermore, accessory or adaptor molecules can be involved in protein binding to the 19S regulator of the 26S proteasome, known as proteasomal Ub receptors. In *S*. *cerevisiae*, there are several Ub receptors that can support protein degradation by the proteasome. Two of these receptors, Rpn10 (S5a in humans) and Rpn13, are stable subunits of the 26S proteasome [59]. The others are UV excision repair protein Rad23 (a repressor protein) and DNA-damage inducible protein1 (Ddi1), which reversibly bind to the 19S regulatory particle where it functions as a receptor to ‘shuttle’ ubiquitinated proteins into the proteasome’s catalytic core for degradation [58]. SerpinB2 does not contain a known UBD as assessed by sequence homology searching but in the context of SerpinB2 interaction with PSMβ1 [40,41] and its non-covalent association with Ub, it is possible that SerpinB2 acts as a co-factor for Ub, assisting ubiquitinated proteins to the proteasome.

## Conclusions

Together, these data suggest that SerpinB2 modulates protein degradation capacity and protein aggregation, both key components in the cellular proteostasis network. We demonstrate the ability of SerpinB2 to protect cells from proteotoxicity associated with protein misfolding and proteostasis dysfunction. We cannot rule out that the cytoprotective mechanism is, at least in part, through its classical serpin inhibitory mechanism. However, our data suggest that SerpinB2 may mediate its observed cytoprotective effect by modulating protein degradation pathways promoting the formation of large inclusion bodies and the prevention of smaller, potentially more toxic oligomeric aggregates. While the precise mechanism remains to be elucidated, SerpinB2 may promote large inclusion body formation by a direct interaction with misfolded proteins and via a functional interaction with the UPS.

## Supporting Information

S1 Fig Flow cytometry analyses. These show representative gating of **(Figure A)** mCherry_(660_20 green)_/GFP_(515_20 blue)_ positive cells (Q2) to select cells positive for both mCherry (Httex1polyQ expression) and GFP (surrogate for SerpinB2 expression), and **(Figure B)** and cell viability analysis by SytoxRed_(660_20 red)_ exclusion of this population.
(PDF)

S2 Fig Huntingtin co-localization with stress granule marker TIA-1. WT MEFs were transiently transfected with mutant Htt_ex1_46Q-mcherry fusions and then incubated for 48–72 h and TIA-1 detected using mouse monoclonal anti-TIA-1 antibody (Santa Cruz; sc-365349) followed by goat anti-mouse IgG Alexa Fluor488-conjugated secondary antibody (Life technologies; A11001). Mouse IgG1 monoclonal antibody (Chemicon, Australia; MABC002) was used as an isotype control (data not shown). Cells were then imaged using laser scanning confocal microscopy. TIA-1 co-localizes to Htt inclusions but not to smaller (< 2 μm) foci.
(PDF)

S3 Fig Huntingtin co-localizes with autophagosome marker LC3-GFP. WT MEFs were transiently co-transfected with LC3-GFP and mutant Htt_ex1_46Q-mcherry fusions and then incubated for 48–72 h. Cells were then imaged using laser scanning confocal microscopy. LC3-GFP co-localizes to smaller (< 2 μm) Htt foci but not to Htt inclusions.
(PDF)

S4 Fig Huntingtin does not co-localize with SerpinB2. WT MEFs were transiently transfected for 48–72 h with Htt_ex1_46Q-mcherry expression vector as described in Methods, prior to fixation with 4% paraformaldehyde followed by permeabilization with 0.1% triton X-100 and blocking with 5% FBS, 1% BSA, 0.1% triton X-100 in PBS. **(Figure A)** SerpinB2 was detected using in-house affinity-purified rabbit anti-mouse serpinB2 polyclonal antibody (2 μg/ml; diluted in 1% BSA, 0.1% triton X-100 in PBS) (Schroder et al., unpublished data) followed by goat anti-rabbit IgG Alexa Fluor488-conjugated secondary antibody (ABCAM; ab181448 1:500 dilution). **(Figure B)** Rabbit IgG antibody (2 μg/ml; ABCAM; ab171870), used as an isotype control, shows specificity of the serpinB2 antibody. Cells were then imaged using laser scanning confocal microscopy. SerpinB2 does not co-localize to Htt inclusions or to smaller (< 2 μm) foci.
(PDF)

S5 Fig SerpinB2 protects cells from overexpression and transduction induced toxicity. Viability of SerpinB2^-/-^ MEFS **(Figure A)**, or SerpinB2^-/-^ MEFS transduced with pMIG control empty vector (vector) **(Figure B)**, or SerpinB2^-/-^ MEFS transduced with pMIG-SerpinB2 vector (WT rescue) **(Figure C)** at 48 h following transfection with lipofectamine alone (Ctl), or Htt_ex1_25Q-mCherry (Htt25Q), Htt_ex1_46Q-mCherry (Htt46Q) or mCherry expression vectors. Data represent mean percentage of viable cells (as measured by SytoxRed exclusion and flow cytometry) normalized to lipofectamine only controls (n = 3 ± SEM).
(PDF)

S6 Fig SerpinB2 abolishes Aβ_1–40_ peptide fibril formation *in vitro*. Aβ_1–40_ aggregation was followed by changes in thioflavin-T fluorescence (490 nm) over time in the absence (control) or presence of SerpinB2, SOD1 (negative control) or αB-crystallin (positive control). Data represent mean fluorescence intensity with background controls subtracted (n = 2 of a representative experiment).
(PDF)
