# Supplementary figures and images for: SerpinB2 (PAI-2) Modulates Proteostasis via Binding Misfolded Proteins and Promotion of Cytoprotective Inclusion Formation
Source: PLoS One. 2015 Jun 17;10(6):e0130136. doi: 10.1371/journal.pone.0130136 (PMC4470917; doi:10.1371/journal.pone.0130136)

Supplementary Figure 1

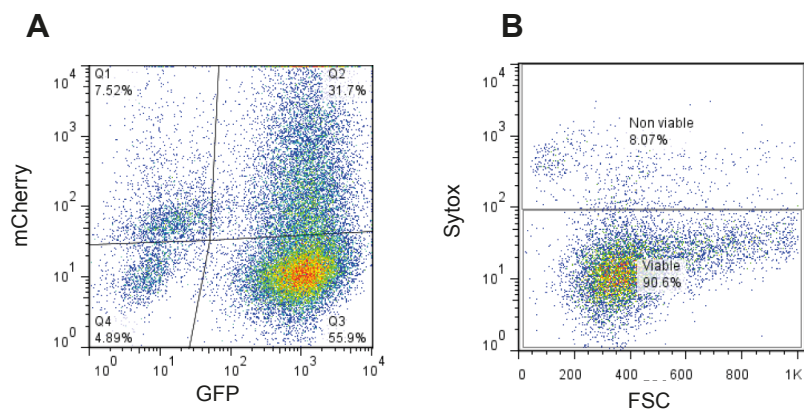

Supplement: S1 Fig — These show representative gating of (Figure A) mCherry(660_20 green)/GFP(515_20 blue) positive cells (Q2) to select cells positive for both mCherry (Httex1polyQ expression) and GFP (surrogate for SerpinB2 expression), and (Figure B) and cell viability analysis by SytoxRed(660_20 red) exclusion of this population. (PDF) [file pone.0130136.s001.pdf]

## Supplementary Figure 2

Htt<sub>ex1</sub>46Q

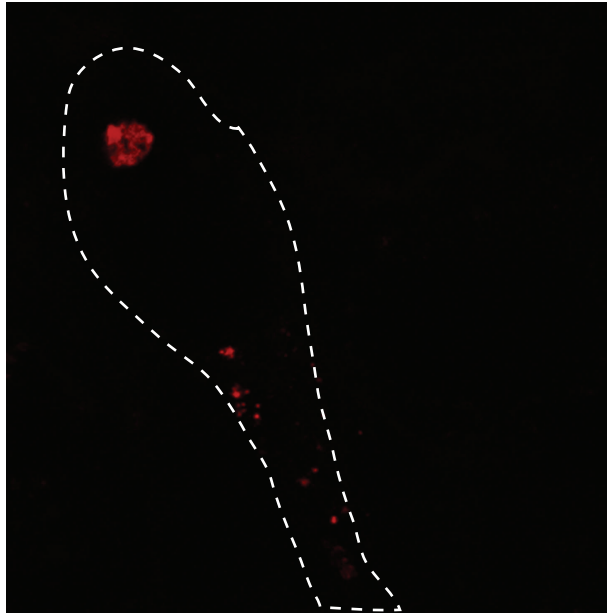

TIA-1

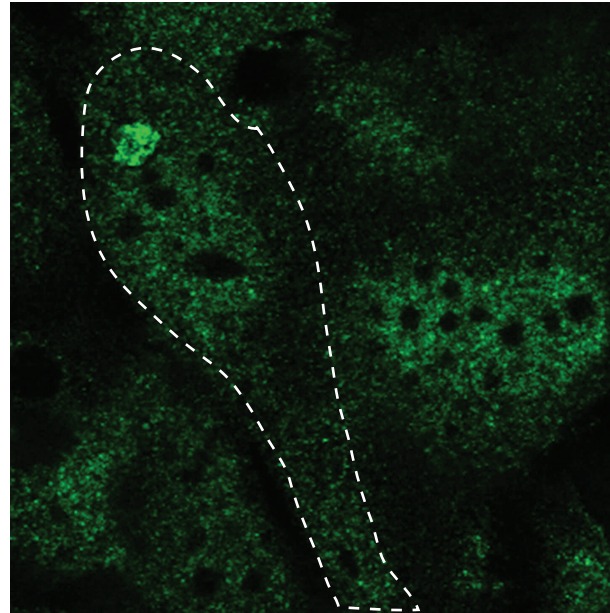

Merge

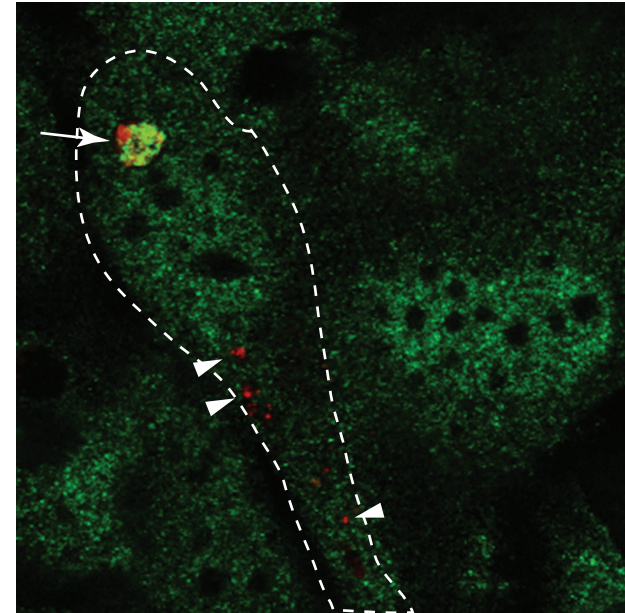

Supplement: S2 Fig — WT MEFs were transiently transfected with mutant Httex146Q-mcherry fusions and then incubated for 48–72 h and TIA-1 detected using mouse monoclonal anti-TIA-1 antibody (Santa Cruz; sc-365349) followed by goat anti-mouse IgG Alexa Fluor488-conjugated secondary antibody (Life technologies; A11001). Mouse IgG1 monoclonal antibody (Chemicon, Australia; MABC002) was used as an isotype control (data not shown). Cells were then imaged using laser scanning confocal microscopy. TIA-1 co-localizes to Htt inclusions but not to smaller (< 2 μm) foci. (PDF) [file pone.0130136.s002.pdf]

## Supplementary Figure 3

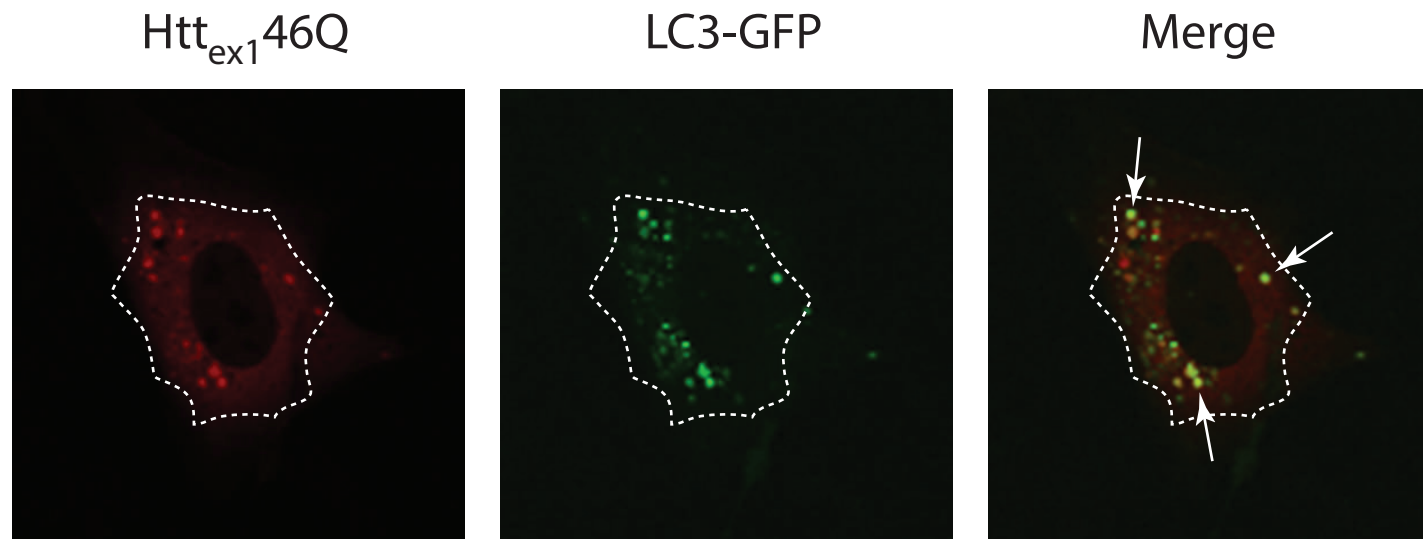

Supplement: S3 Fig — WT MEFs were transiently co-transfected with LC3-GFP and mutant Httex146Q-mcherry fusions and then incubated for 48–72 h. Cells were then imaged using laser scanning confocal microscopy. LC3-GFP co-localizes to smaller (< 2 μm) Htt foci but not to Htt inclusions. (PDF) [file pone.0130136.s003.pdf]

## Supplementary Figure 4

A

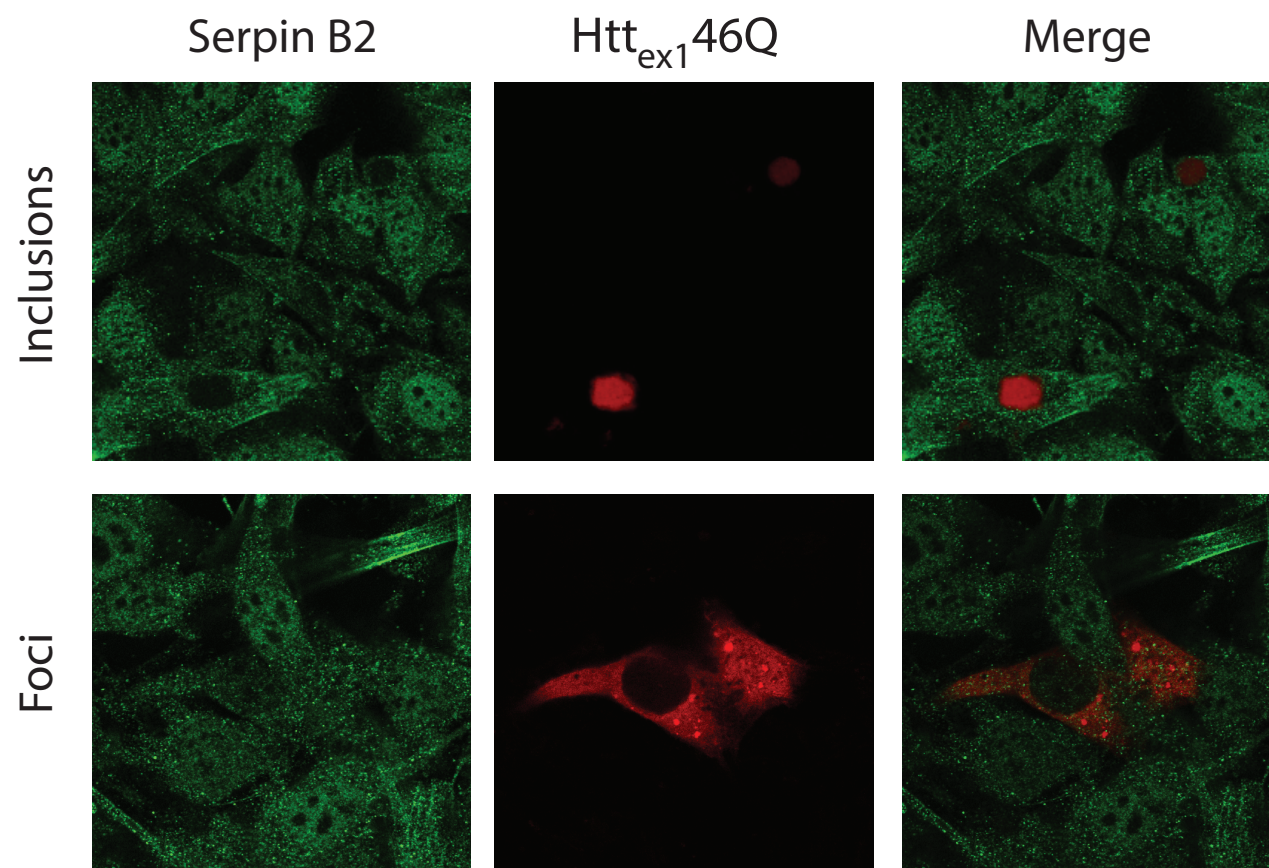

B

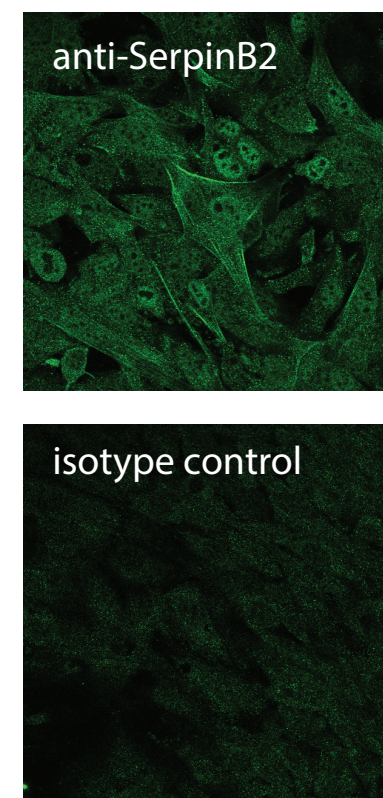

Supplement: S4 Fig — WT MEFs were transiently transfected for 48–72 h with Httex146Q-mcherry expression vector as described in Methods, prior to fixation with 4% paraformaldehyde followed by permeabilization with 0.1% triton X-100 and blocking with 5% FBS, 1% BSA, 0.1% triton X-100 in PBS. (Figure A) SerpinB2 was detected using in-house affinity-purified rabbit anti-mouse serpinB2 polyclonal antibody (2 μg/ml; diluted in 1% BSA, 0.1% triton X-100 in PBS) (Schroder et al., unpublished data) followed by goat anti-rabbit IgG Alexa Fluor488-conjugated secondary antibody (ABCAM; ab181448 1:500 dilution). (Figure B) Rabbit IgG antibody (2 μg/ml; ABCAM; ab171870), used as an isotype control, shows specificity of the serpinB2 antibody. Cells were then imaged using laser scanning confocal microscopy. SerpinB2 does not co-localize to Htt inclusions or to smaller (< 2 μm) foci. (PDF) [file pone.0130136.s004.pdf]

# Supplementary Figure 5

A

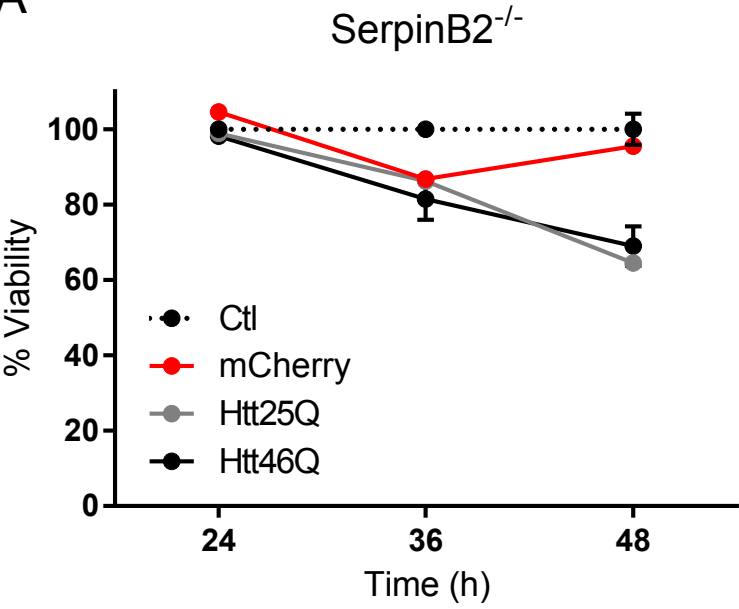

B

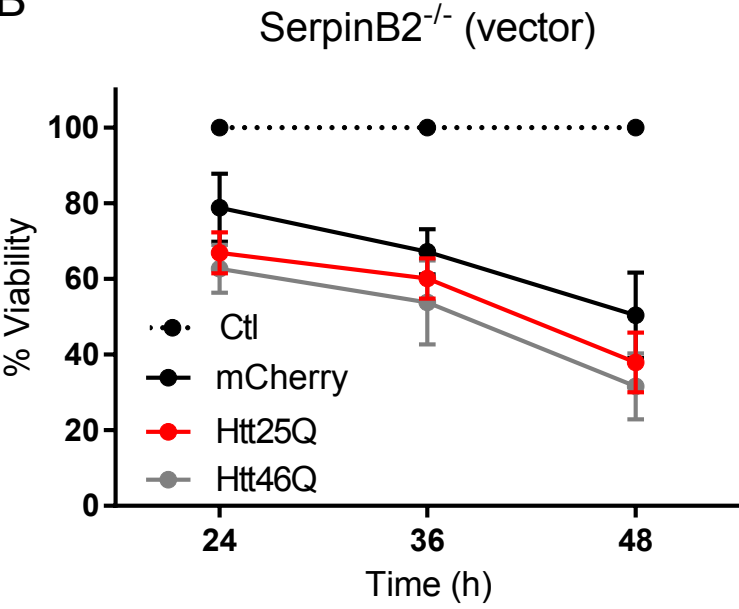

C

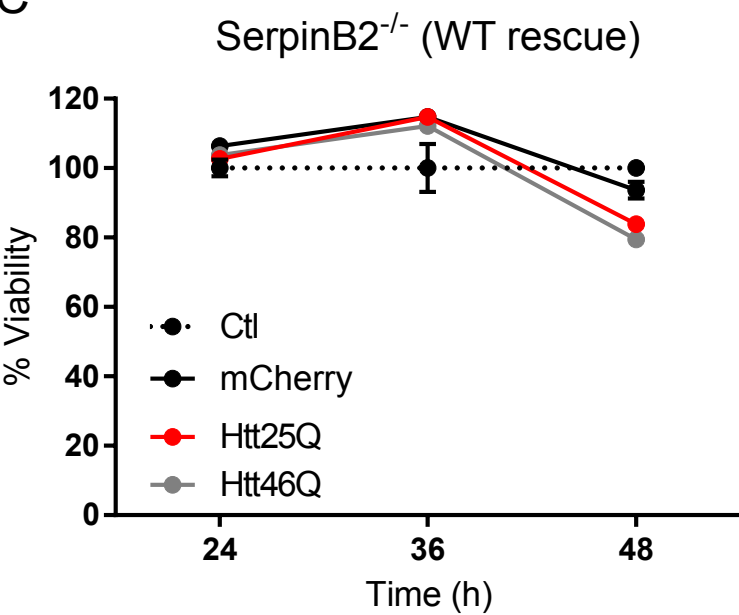

Supplement: S5 Fig — Viability of SerpinB2-/- MEFS (Figure A), or SerpinB2-/- MEFS transduced with pMIG control empty vector (vector) (Figure B), or SerpinB2-/- MEFS transduced with pMIG-SerpinB2 vector (WT rescue) (Figure C) at 48 h following transfection with lipofectamine alone (Ctl), or Httex125Q-mCherry (Htt25Q), Httex146Q-mCherry (Htt46Q) or mCherry expression vectors. Data represent mean percentage of viable cells (as measured by SytoxRed exclusion and flow cytometry) normalized to lipofectamine only controls (n = 3 ± SEM). (PDF) [file pone.0130136.s005.pdf]

**Supplementary Figure 6**

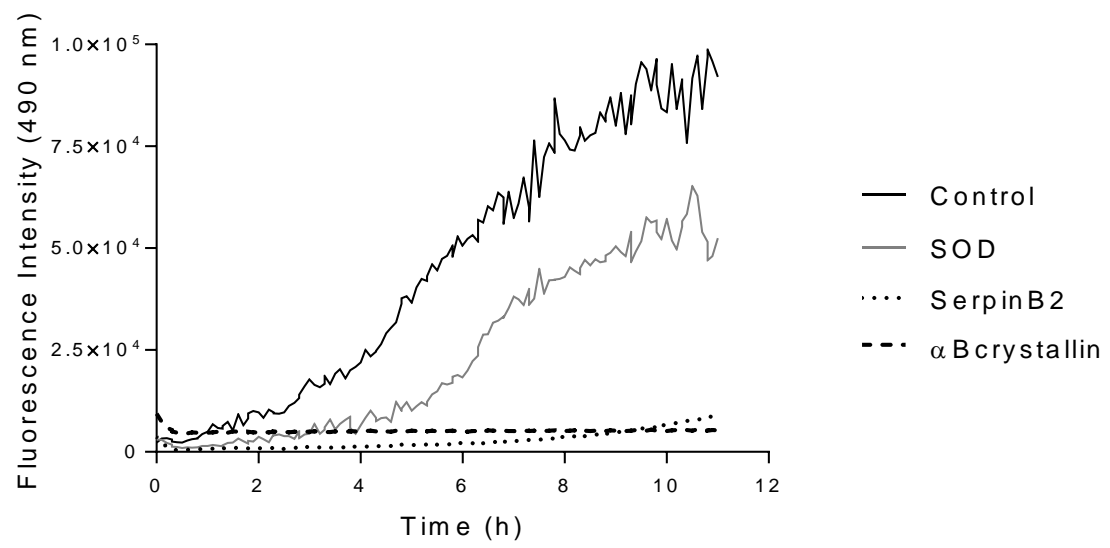

Supplement: S6 Fig — Aβ1–40 aggregation was followed by changes in thioflavin-T fluorescence (490 nm) over time in the absence (control) or presence of SerpinB2, SOD1 (negative control) or αB-crystallin (positive control). Data represent mean fluorescence intensity with background controls subtracted (n = 2 of a representative experiment). (PDF) [file pone.0130136.s006.pdf]
